# Antibiotic use practices of pharmacy staff: a cross-sectional study in Saint Petersburg, the Russian Federation

**DOI:** 10.1186/s40360-017-0116-y

**Published:** 2017-02-14

**Authors:** Tatiana Belkina, Natalia Duvanova, Julia Karbovskaja, Jurjen Duintjer Tebbens, Jiri Vlcek

**Affiliations:** 1 Department of Social and Clinical Pharmacy, Faculty of Pharmacy in Hradec Kralove, Charles University, Heyrovskeho 1203, Hradec Kralove, 50005 Czech Republic; 2 Department of Technology of Dosage Forms, Saint Petersburg State Chemical - Pharmaceutical Academy, Prof. Popova Str. 14, Saint Petersburg, 197376 Russian Federation; 3 Department of Biophysics and Physical Chemistry, Faculty of Pharmacy in Hradec Kralove, Charles University, Heyrovskeho 1203, Hradec Kralove, 50005 Czech Republic

**Keywords:** Antibiotic use, Pharmacists, Antimicrobial resistance

## Abstract

**Background:**

Non-prescription access to antimicrobials is common, and self-prescribing is increasingly popular in Russian society. The aim of this study was to assess the attitudes of community pharmacists regarding antibiotic use and self-medication.

**Methods:**

We conducted a cross-sectional study from September-December 2015 of community pharmacists in the Saint-Petersburg and Leningrad region, Russia. A self-administered questionnaire was used to assess antibiotic use and self-medication practices. The data were analysed using logistic regression and Pearson chi-squared tests.

**Results:**

Of the 316 pharmacists (77.07%) who completed the questionnaire, 230 (72.8%) self-medicated with antibiotics. Antibiotics were mostly used to self-treat upper (53.3%) and lower respiratory tract infections (19.3%), relying on their own knowledge (81.5%), previous treatment experience (49%) and patients’ prescriptions (17%). The most commonly used antibiotics were macrolides (33.2%). Characteristics such as age, education and experience were related to antibiotic use and self-medication.

**Conclusions:**

The study confirmed that self-prescription of antibiotics is a common practice amongst pharmacists in Saint Petersburg and also identified personal and professional characteristics of pharmacists strongly associated with self-medication.

**Electronic supplementary material:**

The online version of this article (doi:10.1186/s40360-017-0116-y) contains supplementary material, which is available to authorized users.

## Background

In the past decade, the worldwide consumption of antibiotic drugs has increased substantially. Russia, along with Brazil, India, China, and South Africa, accounts for 76% of the overall increase in the global consumption of antibiotics [1]. Non-prescription access to antimicrobials, including antituberculosis drugs, is common, and self-prescribing has become increasingly popular in Russian society [2]. Conditions that require prescriptions for the dispensation of antibiotics are not explicitly defined in the legislation of the Russian Federation. This leads to arbitrary attitudes toward antibiotics among health professionals, especially pharmacists, whose primary role in dispensing over-the-counter antibiotics offsets with imperfect enforcement. Moreover, in an environment with a relatively low level of public trust in physicians and the lack of formal need for a doctor’s office visit, pharmacists have become the main alternative for patients not only in providing proper counselling but in functioning as a substitute for physicians in antibiotic selection, the administration of antibiotic regimens and the course of therapy [3].

The purpose of this article is to explore pharmacists’ approach to antibiotic treatment, including antibiotic choice, and to assess their knowledge of and attitudes toward antimicrobial resistance to define ways to prevent the practice of dispensation without prescriptions.

## Methods

### Study setting and population

The study was conducted from September-December 2015 in community pharmacies in the Saint Petersburg and Leningrad region, Russia. The sample comprised 63 pharmacies and 316 pharmacists from one of the largest pharmacy chains.

### Measures

A self-administered questionnaire was adapted from a previous study conducted by our research group [4]. The questionnaire included close-ended (yes/no, single choice and multiple choice) and open-ended questions (an English translation is available in Additional file 1). Denoting the sixteen questions from Q1 to Q16, they can be grouped into the following categories: attitudes and behaviours towards antibiotic use and self-medication (Q1, Q2, Q3, Q7, Q11, Q12); information on the types of involved diseases, antibiotics and side effects (Q3 partially, Q4, Q9); knowledge of antibiotic use and resistance and source of antibiotic knowledge (Q5, Q6, Q8, Q10); and personal and professional information (Q13 – Q16).

### Statistical analysis

To evaluate the influence of attitudes, behaviour, knowledge and demographics on self-medication with antibiotics, univariate logistic regression analyses were performed. Self-medication occurred when the respondents indicated that they purchased antibiotics for themselves (or their children) without a physician’s prescription but based on their own knowledge or on recommendations of friends (see part two of Q2). This defined the dichotomous dependent variable. The independent variables were, respectively, the outcomes of questions Q1, Q3, Q5, Q6, Q11, Q12, Q13, Q14, Q15 and Q16. We did not include variables related to information on the types of involved diseases, antibiotics and side effects (Q3, Q4, Q9), as they contained too many outcomes, and we also did not include variables with essentially only one outcome (Q8, Q10, Q13). For most of the included questions, all respondents provided answers; however, questions Q14 to Q16 had a percentage of missing values of 0.3% (1 answer) each, and Q7 was missing 3.2% (10 answers). In a few cases, multiple answers to the same question were provided; the highest score (e.g., highest education, highest age, best knowledge) was then used for the regression analysis. For question Q6, however, there were too many multiple answers to address using this approach. Therefore, Q6 was divided into three separate dichotomous variables with outcomes yes or no: Q6a, Q6b and Q6c.

The results of the regression models were presented as the estimated odds ratios and the corresponding 95% confidence intervals. Hypothesis tests for regression coefficients (Wald tests) were performed and expressed with p values at a significance level of α = 0.05.

We also tested for associations between demographic characteristics (Q14 – Q16) and the remaining independent variables. We used Pearson chi-squared tests with *p* values at a significance level of α = 0.05. To avoid low expectation counts, the outcomes of Q14 – Q16 were recategorized (in clear ways) into two groups. PASW statistical software was used for all analyses (IBM Corporation, Armonk, NY, USA, version 18.0).

## Results

Of the 410 questionnaires distributed, 316 (77.07%) were completed and collected.

The main demographic characteristics of the respondents are summarized in 1. The sample was predominantly female, which is rather typical for Russian pharmacies. The mean age of the respondents was 39.3 years, and almost half of them were 41-60 years. The vast majority of pharmacists had a baccalaureate degree, and nearly half of them had 10 years of experience of working in a pharmacy. Table 1 shows that slightly more than two-thirds of the pharmacists self-medicated.

**Table 1:** Personal and professional characteristics of pharmacists, 2015 (*n =* 316)

Characteristic	No (%) (*N =* 316)
Gender:	
Male	4 (1.27)
Female	312 (98.73)
Age (years):	
<20	1 (0.32)
20–30	100 (31.65)
31–41	65 (20.57)
41–60	143 (45.25)
>60	7 (2.21)
Education:	
Higher pharmaceutical	110 (31.5)
Other higher	33 (9.5)
Vocational pharmaceutical	206 (59.0)
Years of experience in a pharmacy:	
<1	15 (4.75)
1–5	85 (26.90)
6–10	83 (26.26)
>10	133 (42.09)
Source of antibiotics:	
Prescribed	100 (31.65)
Self-medication	216 (68.35)

Other characteristics of the sample that are not displayed in Table 1 are as follows.

About three-quarters of the respondents (72.8%) self-medicated when they were sick. The highest prevalence of self-medication was reported for the age group of those 41-60 years old (45.3%). Young and middle-aged respondents (31-40 years) were more responsible: 22.8% of them went to the doctor, and 20.6% self-medicated. However, the logistic regression analysis, which is addressed in more detail later, did not reveal statistically significant higher probabilities of self-medication for any of these age groups. Only 33% purchased antibiotics using a doctor’s prescription. More than half (61%) of the respondents visited a doctor for an examination and received a prescription from time to time, and 6% of staff pharmacists never bought antibiotics from a prescription. The respondents who did not visit or rarely visited a doctor for prescriptions bought antibiotics based on their knowledge (81.5%) or their friends’ advice (5.0%); analyses of their experiences with previous treatment (49.0%); and prescriptions provided by pharmacy customers (17.0%). Only a small number of respondents (8.0%) considered cost to a significant degree when purchasing medicine.

Antibiotics were mostly used to self-treat upper and low respiratory tract infections (53.3% and 19.3%, respectively). Other conditions addressed with self-medication were dental problems, urogenital infections and respiratory inflammation.

The most commonly used antibiotics were macrolides (33.2%). Azithromycin was predominant in this group and accounted for 81.1% of all self-prescriptions. Semi-synthetic penicillins (30.9%) and fluoroquinolones (15.2%) were other most frequently used groups, followed by tetracyclines (7.8%), second-generation cephalosporins (5.5%), lincosamides (5.4%) and aminoglycosides (2.0%).

The main source of information regarding antibiotics was training sessions (43.2%), patient information leaflets (30.1%) and specific literature (26.7%). More than half of the respondents (66.8%) took their antibiotics according to the information on the leaflet, one-third (31%) took antibiotics in line with their physician’s prescriptions, and the rest (2.2%) stopped using the antibiotic early when their symptoms decreased.

Oral dosage was the most preferred form of antibiotics.

Table 2 shows the impact of pharmacists’ attitudes, behaviour and knowledge on self-medication with antibiotics.

**Table 2:** Factors influencing self-medication with antibiotics based on univariate logistic regression models for individual questions. Reference groups are the outcomes not displayed between brackets after the question

Question (evaluated outcome)	No. (*N =* 316)	*p*-value (Wald-test)	Odds ratio	95% confidence interval	
When you feel ill (consult a physician)	86	0.001*	0.405	0.232	– 0.707
Have you taken antibiotics in the past 6 months (no)	175	0.727	0.915	0.555	– 1.508
Antibiotic use:					
(Stop after symptoms decreased)	8	0.727	0.759	0.162	– 3.560
(As per leaflet)	243	0.005*	2.088	1.250	– 3.488
Source of new information about antibiotics:					
- Training sessions (no)	227	0.014*	0.517	0.305	– 0.877
- Special literature (no)	140	0.002*	0.441	0.260	– 0.747
- PIL^a^ (no)	158	0.800	1.066	0.649	– 1.750
Preferable form (injections)	285	0.207	0.571	0.24	– 1.362
Use of probiotics (no)	285	0.674	0.844	0.384	– 1.856
Attitude toward antibiotic therapy:					
(Against)	10	0.964	0.955	0.126	– 7.229
(Extreme cases)	295	0.612	0.714	0.194	– 2.624

*p < 0.05

^a^ *PIL* Patient information leaflet

The following associations were found to be statistically significant. When feeling ill, those who consulted a physician had a lower probability (OR 0.41) of self-medication than those who self-medicated, which is self-evident. Concerning the way antibiotics were used, those acting according to patient leaflets had a higher chance (OR 2.09) of self-medication than those who used antibiotics as prescribed by the physician. Interestingly, pharmacists who did not obtain new information on antibiotics through educational paths or specific literature exhibited a substantially decreased probability of self-medication (OR 0.57 and OR 0.44, respectively). Although not displayed in Table 2, the logistic regression analysis indicated a strong association of self-medication with all levels of education compared to those with basic (non-pharmaceutical) vocational education, with the following odds ratios: higher pharmaceutical (OR 5.201, CI 95% 1.87–14.42), other higher education (OR 3.781, CI 95% 1.12 -12.79) and vocational pharmaceutical (OR 3.613, CI 95% 1.36–9.59).

The outcomes of questions Q8 (knowledge about side effects), Q10 (knowledge about influence on normal flora) and Q13 (gender) were essentially identical (up to less than one percent) for all respondents. This similarity is explained by the fact that the respondents were with a common pharmaceutical background and 98.73% of the respondents were females.

Pearson chi-squared tests revealed no association between personal characteristics (age, education and experience) and self-medication or antibiotic use (Table 3). By contrast, variables related to the source of new information about antibiotics were shown to be significantly associated with personal characteristics.

**Table 3:** Association of demographic characteristics with attitudes towards antibiotic use

Statement	*p*- value			
	No.	Age	Education	Experience
	(*N =* 316)			
Self-medicate	230	0.946	0.565	0.123
Antibiotics taken in the past 6 months	141	0.787	0.323	0.807
Stopped taking antibiotics after symptoms decreased	8	0.779	0.34	0.382
Source of new information about antibiotics:				
- Training sessions	227	0.309	0.889	0.047*
- Special literature	140	0.318	0.003*	0.051
- PIL^a^	158	0.044*	0.465	0.195
Taking probiotics during/after treatment with antibiotics	283	0.869	0.805	0.15
Attitude toward antibiotics	298	0.683	0.622	0.633

*p < 0.05

^a^PIL: Patient information leaflet

## Discussion

The results of our study show that the practice of self-prescribing antimicrobials is extremely popular amongst pharmacists in Saint Petersburg. We suggest that the main contributing factor of this high prevalence is pharmacists’ easy access to antibiotics, since a prescription for the dispensed antibiotics is neither required nor controlled by authorities during inspections.

Data on outpatient antibiotic use reported by the European Surveillance of Antimicrobial Consumption suggested that in 2009, Russia was the third largest outpatient consumer of antibiotics in Europe when consumption was expressed as the number of packages per 1000 inhabitants per day [5].

Another study by Stratchounski et al. also confirmed that the Russian population is inclined to stock antibiotics in home medicine cabinets for further uncontrolled and unsupervised use [6].

While Russian physicians are aware of antibiotic resistance and are concerned by the over-the-counter sale of antibiotics [7], pharmacists promote an inappropriate use of medications by over-the-counter dispensation. This finding can be explained by the lack of a culture in which drugs are dispensed/purchased strictly based on prescriptions. Physicians often do not write prescriptions or they provide their recommendations, including the use of antibacterial drugs, on a regular sheet of paper that may, in the best case, have a physician’s seal.

Our findings indicate that antibiotics were commonly used by pharmacists to treat upper respiratory tract infections, which raises concern regarding the potential misbelief that antibiotics can treat and eradicate infections regardless of its origin [8], as well as a lack of professional knowledge of antibiotics that largely do not act on acute cough and colds [9]. Nevertheless, the results showed that educational strategies aimed at improving professional knowledge through training sessions or specific literature could have an undesired effect on pharmacists’ intention to self-medicate. Some pharmacists explained this finding by saying that participating in trainings and receiving additional information about medicines, especially from medical representatives of drug companies, contributes to improving their professional skills and saves time because it removes the need to visit a doctor.

The most popular group of antibiotics used by the respondents was macrolides. In contrast, a study on the outpatient use of systemic antimicrobials in 24 different regions of Russia reported that in 23 regions (including Saint Petersburg), broad-spectrum penicillins were the most frequently used antimicrobials. It can be assumed that there is a significant difference in the outpatient consumption of systemic antimicrobials in different regions of Russia [10]. In some regions, older agents with unfavourable safety profiles are widely administered, whereas in other regions, newer agents are used more frequently.

Diseases such as tuberculosis, gonorrhoea, malaria and childhood ear infections are now more difficult to treat than they were a few decades ago [11]. High levels of resistance to ciprofloxacin, penicillin G, azithromycin, spectinomycin and carbapenems have been reported in Russia, as a potential result of the inappropriate use of antimicrobials [12, 13].

The widespread practice of self-medication in Russia is a result of the insufficient coverage of drug programmes and the existing problems with access to medical care. The absence of a medication reimbursement system, meaning medicines remain out-of-pocket payments, is another reason for self-medication [14], significantly affecting the prices of medicines and their availability to the public [15]. In Russia, only predetermined categories of the population receive free medication within the ONLS (Population Drug Coverage) and DLO (Extensive Drug Coverage) programmes. However, it is rather difficult for patients to obtain preferred prescriptions, as the procedure for issuing prescriptions is time-consuming and pharmacies have insufficient quantities of medicine (deficit, supply disruptions).

This study is limited by the fact that the data were self-reported, and there is thus a possibility that the participants over-reported socially desirable behaviours or under-reported socially undesirable behaviours. There were no mechanisms that objectively assessed the honesty of the participants’ answers to the survey questions. The absence of identifying data on the questionnaire sheets and the confidential nature of the study would tend to minimize this bias.

The role of pharmacists in encouraging the prudent use of antimicrobials is clearly vital. However, the practice of dispensing non-prescribed antibiotics continues to be widespread in some European countries [16–18]. Other research should focus on finding the causes of this condition. An option is to evaluate the knowledge and attitudes of pharmacists towards the antibiotic resistance and the basic principles of antibiotic therapy. Similarly, this could be an analogy with the project conducted among the Czech pharmacists, whose attitudes towards the use of generic substitution significantly reflected the level of the knowledge on generic medications [19].

## Conclusions

Pharmacy employees must understand the rules, orders, and other relevant information on how to dispense antibiotics. However, most of these pharmacists do not follow these regulations. It is suggested that a well-planned, organized and structured educational programme for doctors and pharmacists should be implemented to improve the appropriate use of antibiotics.

## Additional file

Additional file 1: Questionnaire “Use of antibiotics by pharmacy employees”. (DOCX 17 kb)
