# Supplementary material for: Antibiotic use practices of pharmacy staff: a cross-sectional study in Saint Petersburg, the Russian Federation
Source: BMC Pharmacol Toxicol. 2017 Feb 14;18:11. doi: 10.1186/s40360-017-0116-y (PMC5307805; doi:10.1186/s40360-017-0116-y)
Supplement: Additional file 1: — Questionnaire “Use of antibiotics by pharmacy employees”. (DOCX 17 kb) [file 40360_2017_116_MOESM1_ESM.docx]

**Additional file**

**Questionnaire**

**Use of antibiotics by pharmacy employees**

*Dear colleague, please answer the following questions.*

*You can choose more than one option.*

*Mark your answers with an [X] or [V].*

| Q1. | When you’re falling ill:  You go to the doctor [ ]  You self-medicate [ ] |
| --- | --- |
| Q2. | When you or one of your family members uses antibiotics, usually you buy it:   1. **According to a doctor's prescription:**   Always [ ]  Never [ ]  Sometimes [ ]  **2.** **Without a doctor's prescription**:  According to your own knowledge [ ]  According to your experiences with previous treatment [ ]  According to analyses of the instructions with which customers come to the pharmacy [ ]  According to a friend’s advice [ ]  According to the cost of the medicine [ ] |
| Q3. | Have you or one of you family member taken any antibiotic in the past 6 months:  Yes [ ] How many times ________­­­­­­­­­­­­­­______________  No [ ]   - If «Yes», what was the reason:   Upper respiratory tract infection [ ]  Lower respiratory tract infection [ ]  Dental infection [ ]  After surgery [ ]  Gastrointestinal infection [ ]  Gynaecological inflammation [ ]  Bone and diarthrosis infection [ ]  Skin and soft tissue infection [ ]  Urogenital infection [ ]  I have a chronic infectious disease [ ] |
| Q4. | Please name the antibiotics that you or a family member has taken in the past 6 months and **how many times**:  ___________________________________________________________________ |
| Q5. | Antibiotic use:  I stop taking antibiotics when feeling better [ ]  I take antibiotics as prescribed by the physician [ ]  I take antibiotics as per their instructions for medical use [ ] |
| Q6. | You receive information about antibiotics through:  Training sessions [ ]  Relevant medical literature [ ]  Patient information leaflet (PIL) [ ] |
| Q7. | You prefer:  Oral form [ ] Injection form [ ] |
| Q8. | Are you aware of the unexpected side effects that can occur with antibiotic treatment:  Yes [ ] No [ ] |
| Q9. | Which side effects did you experience during your antibiotic treatment:  Diarrhoea [ ]  Constipation [ ]  Vomiting [ ]  Nausea [ ]  Dyspepsia [ ]  Skin manifestations [ ]  Myxedema [ ]  Mucositis [ ]  Asthenia [ ]  Hyperhidrosis [ ]  Tachycardia/bradycardia [ ]  Blood pressure changes [ ]  Other (please, name)__________________________________________  _____________________________________________________________________  I did not have side effects [ ] |
| Q10. | Are you aware that antibiotics kill off normal microflora:  Yes [ ] No [ ] |
| Q11. | Do you take any probiotics during/after AB treatment:  Yes [ ] No [ ] |
| Q12. | Your attitude toward antibiotic therapy:  Antibiotics are my first choice of medicine [ ]  I take antibiotics only in extreme cases [ ]  I am totally against antibiotics [ ] |
| Q13. | Gender： Male [ ] Female [ ] |
| Q14. | Your age：  < 20 years [ ] 20-30 years [ ]  31-40 years [ ] 41-60 years [ ] > 60 years [ ] |
| Q15. | Your education:  Higher pharmaceutical education [ ]  Other higher [ ] Please state in which field ___________________  Vocational pharmaceutical degree [ ]  Other vocational degree [ ] Please state in which field ___________________ |
| Q16. | How long have you been working in the pharmacy:  < 1 year [ ] 1-5 years [ ] 6-10 years [ ] > 10 years [ ] |

Date _______ __________ 20____

day month year

Place _____________________________________________________

**Thank you for your participation!**
